# Effect of pulmonary rehabilitation on all-cause mortality in patients with chronic respiratory disease: a retrospective cohort study in an Australian teaching hospital

**DOI:** 10.1186/s12890-024-03319-9

**Published:** 2024-10-10

**Authors:** Vahid Sharifi, Danny J. Brazzale, Christine F. McDonald, Catherine J. Hill, Chris Michael, Warren R. Ruehland, David J. Berlowitz

**Affiliations:** 1 Institute for Breathing and Sleep, Austin Hospital, Heidelberg, VIC Australia; 2 Department of Respiratory and Sleep Medicine, Austin Hospital, Heidelberg, VIC Australia; 3 Department of Physiotherapy, Austin Hospital, Heidelberg, VIC Australia; 4 Faculty of Medicine, Dentistry and Health Sciences, The University of Melbourne, Melbourne, VIC Australia

**Keywords:** Asthma, Chronic respiratory disease, COPD, ILD, Mortality, Pulmonary rehabilitation, Spirometry, Survival analysis

## Abstract

**Background:**

Pulmonary rehabilitation (PR) is widely recommended for short-term benefits in chronic respiratory diseases, yet long-term outcomes remain uncertain. This retrospective cohort study addresses this gap, comparing 20-year mortality rates between PR participants and matched controls, and hypothesizing that the short-term benefits of PR contribute to improved long-term survival.

**Methods:**

The 20-year mortality of stable chronic respiratory patients who participated in an outpatient PR program was compared with a matched control group based on the type of lung disease. Demographic and clinical variables, and the dates of deaths, were extracted and compared between two groups with two sample t-test and chi-square tests. Kaplan-Meier plots and Cox regression analyses were employed to evaluate survival differences.

**Results:**

Between 2000 and 2002, 238 individuals enrolled in a pulmonary rehabilitation (PR) program (58% male, mean age ± SD: 69 ± 8 years, mean FEV_1_% predicted ± SD: 46 ± 21%). An equal number of people with comparable lung disease were selected as controls (88% COPD, 5% ILD). Controls had lower FEV_1_% predicted values (mean ± SD: 39 ± 17%, *P* < 0.001), smoked more (mean ± SD: 48 ± 35 pack-years, *P* = 0.032), and no differences in age, BMI, sex, and Index of Relative Socio-economic Advantage and Disadvantage (IRSAD). Median (IQR) follow-up time was 68 months (34–123), with 371 (78%) deaths. Univariable (HR = 1.71, *p* < 0.001) and multivariable (HR = 1.64, *p* < 0.001) Cox regression found higher mortality risk in controls. Subgroup analysis for COPD replicated these findings (HR = 1.70, *P* < 0.001).

**Discussion:**

Despite some methodological limitations, our study suggests that clinically stable patients with chronic respiratory disease who undertake PR may have lower mortality than matched controls.

**Trial registration:**

Retrospectively registered.

## Background

Pulmonary rehabilitation (PR) is widely recommended in the management of patients with chronic respiratory disease including chronic obstructive pulmonary disease (COPD), asthma, interstitial lung disease (ILD) and bronchiectasis [1–6]. There is consistent evidence of short-term benefit in outcomes of dyspnoea, health related quality of life, and functional exercise capacity in patients with stable disease who undertake PR [7–10]. There is less certainty regarding longer term outcomes of morbidity and mortality with most studies underpowered for these outcomes, follow up rarely being beyond 12 months, and differing outcomes between patients with stable disease (COPD) who commence PR compared with commencement in the peri-exacerbation period. Lindenauer et al. (2020) [11] conducted a meta-analysis demonstrating increased one-year survival in COPD patients who began PR within three months of discharge. Similarly, a systematic review by Hakamy et al. (2017) [12] reported one-year mortality benefits in stable COPD patients, though significant benefits were not observed at three years. In addition, Nolan et al. (2021) [13] found an association between increased one-year all-cause mortality and PR incompletion in patients with Idiopathic pulmonary fibrosis (IPF) in an observational cohort study, highlighting a potential risk for those who do not complete PR. However, an earlier meta-analysis of randomized controlled trials [14] highlighted the low quality of evidence supporting these mortality reductions, suggesting that the observed benefits may not be as robust as they appear and underlining the need for further high-quality research.

The considerable and significant short-term benefits of PR in people with stable respiratory disease preclude new randomised controlled trials of PR [7]. This leaves non-controlled or quasi-controlled methodology to estimate the effect of PR on survival in patients with chronic respiratory disease [12]. Data from such trials suggest there may be a difference in survival between those who have participated in PR versus those who have not. However, confidence in these results is limited by study methodology. The current study aimed to compare the 20-year mortality rate of stable outpatients with chronic respiratory disease who undertook a supervised PR program with a matched control group of those who did not. We hypothesized that the short-term benefits of PR, including improved physical conditioning and reduced symptoms, could cumulatively contribute to better long-term health management and potentially influence survival over an extended period.

## Methods

We undertook a retrospective cohort study to analyze the long-term mortality of individuals who enrolled in outpatient PR between January 2000 and December 2002 at a tertiary hospital in Melbourne, Australia. We established a control group, matched by type of chronic lung disease, from individuals who underwent a lung function test at the Respiratory Laboratory at the same tertiary hospital during the same timeframe.

Data were obtained from a prospectively maintained PR database that included all patients referred to the outpatient PR program, completed an initial assessment, and attended at least one PR session. Patients were usually referred by a respiratory specialist after being diagnosed with a chronic respiratory disease. They were enrolled in the program when clinically stable (at least four weeks after an exacerbation or hospital admission) and remained symptomatic (Medical Research Council score ≥ 2) despite optimal medical therapy. Patients with significant comorbidities which would interfere with the ability or safety to exercise were excluded from the program. The PR program consisted of baseline assessment followed by twice weekly attendance at the hospital for six weeks with content provided according to consensus guidelines [2]. Each session included components of supervised exercise and education, lasting for two hours and had been published previously [15, 16]. Exercise training included thoracic mobilization exercises and stretching in addition to 15 min each of individualized walking (treadmill) and cycling and strengthening exercises. The treadmill exercise intensity was initially set at 70% of the baseline 6MWT speed and progressed weekly by 0.25 to 0.5 km/hr guided by the modified BORG scores for perceived dyspnea. The prescription and progression of stationary cycling was based on symptom scores, with patients exercising at an intensity to achieve a modified Borg score of three to four. Lower limb strength training consisted of functional exercises such as step-ups and rising from a stool while upper limb training focused on endurance training (10–20 repetitions) using free weights (1–4 Kg). Supplemental oxygen was provided during training as necessary to achieve oxygen saturation ≥ 88%. All participants were provided with an exercise diary and encouraged to complete a home exercise program that included daily walking. The education component of the PR program was provided by a multidisciplinary team and involved interactive sessions covering topics of lung disease, medications, oxygen therapy, symptom management, airway clearance, nutritional advice, energy conservation, relaxation and anxiety management, coping with chronic illness and social supports.

Patients were identified as “cases” and included for analyses if they (1) had a respiratory disease diagnosis and (2) had a valid spirometry result. For those who had multiple enrolments in PR, only the last one in the specified period was considered and, where multiple spirometry results were available, only the result closest to the initial PR appointment was used. We selected cases from 2000 to 2002 to ensure sufficient outcomes of interest (mortality) in the comparisons. Data on adherence or the number of sessions attended were not recorded, but attendance at the post-program review was documented.

Patients attending the Austin Health Respiratory Laboratory for spirometry between January 2000 and December 2002 were included in the control pool (usual care) if they (1) had a respiratory disease diagnosis, and (2) did not participate in PR at the Austin between 1998 and 2007. If multiple spirometry results were available for controls, only the last one from the specified period was used.

Cases were categorized for primary lung disease, and a frequency match performed to find controls matched for cases in each category. Patients in the control pool were also categorized for primary lung disease and then arranged according to their hospital unique record number (UR). To identify matches for our cases, an equivalent number of patients were selected for each category from the control pool, starting from the uppermost section of the UR-sorted list.

Age was computed in years based on the baseline date (January 1, 2000) for each individual. The severity of lung disease was represented by the recorded FEV_1_ percent predicted, which was extracted from the Austin Health Respiratory Laboratory database. In cases where spirometry results were absent from the respiratory laboratory database, data from the PR database were used.

Other variables such as sex, BMI, and pack-year smoking and current smoking status were extracted from the same lung function report. The Index of Relative Socio-economic Advantage and Disadvantage (IRSAD) for each individual was derived from their postal code, utilizing data from Socio-Economic Indexes for Areas 2001 (SEIFA 2001), accessible on the Australian Bureau of Statistics website. This index was used to determine social and economic wellbeing for each individual. Low values indicate areas of disadvantage and high values indicate areas of advantage.

To determine the date of death (DOD) for both groups, cross-referencing was performed using the Austin Health medical record and an online open-source record (mytribute.com.au) in June 2022. When there was no evidence of death, the date of the last hospital encounter was recorded as the censored date.

Baseline differences were examined between the case group and the matched control group. A two-sample t-test was applied to assess disparities in FEV_1_ percent predicted, age, BMI, IRSAD, and pack-year smoking. Additionally, a chi-squared test was utilized to evaluate discrepancies in sex distribution and current smoking status at baseline.

A Kaplan-Meier plot was generated to contrast the survival probabilities of cases and controls as a function of time. Subsequently, a log-rank test was utilized to assess the significance of the differences observed between the survival curves. Patients without a date of death were not included in the event count (death), but their survival time up to the point of censoring was included in the survival analysis.

Univariable and multivariable Cox regression analyses were conducted to explore the individual associations of PR attendance, age, sex, FEV_1_ percent predicted, BMI, pack-year smoking, current smoking status and IRSAD with mortality, both independently and after accounting for other variables. The concordance index was computed to evaluate the precision of the Cox model. This analysis was repeated for a subset of only COPD patients. A significant level of *p*-value less than 0.05 was adopted, and all analyses were carried out using the Survival package in R version 4.3.1.

## Results

Of the 337 PR enrollments between 2000 and 2002, 238 individuals met all inclusion criteria. The median (IQR) time between spirometry testing and the initial PR appointment was 82 (31 to 287) days. The 238 cases ranged in age from 47 to 92 years with a mean (± SD) of 69.3 (± 7.7) years, a male predominance (*n* = 139, 58.4%) and a mean FEV_1_ (± SD) of 45.9 ± 21.0% predicted. COPD was the most common primary respiratory disorder (*n* = 210, 88.2%), with asthma, ILD and bronchiectasis primarily diagnosed in 5%, 4% and 2% of the cases, respectively. 86% of these patients had a post-program review.

7208 spirometry tests were performed at our hospital between 2000 and 2002 and, after applying inclusion criteria, 238 patients with matching primary diagnosis to cases were selected as the control group. Selected controls were not statistically significantly different in age (mean age ± SD: 69.8 ± 7.9, *p* value = 0.501), or sex (*n* = 155 male, 65.1%, *p* value = 0.157) but had a significantly lower FEV_1_ percent predicted (mean FEV_1_ ± SD: 39.2 ± 17.1% predicted, *p* < 0.001) compared to the PR group. Table 1 provides a comparison of various characteristics between the cases and controls at baseline.

The median (IQR) follow-up time for the study population was 68 months (34 to 123). A total of 371 patients (78%) died during follow-up.

**Table 1:** Baseline characteristics of both cases and controls

Baseline means (SD)	Enrolled PR (*n* = 238)	Did not Enroll PR (*n* = 238)	*p* value
Age at baseline (years)	69.3 (7.7)	69.8 (7.9)	0.501
Sex (male/female)	139/99	155/83	0.157
FEV_1_% predicted	45.9 (21.0)	39.2 (17.1)	< 0.001
BMI (kg/m^2^)	26.9 (6.6)	26.4 (6.8)	0.473
Smoking (Pack-Year)	41.0 (31.7)	47.6 (35.2)	0.032
Current smoker (yes/no)	17/221	30/208	0.046
IRSAD	1020.0 (81.5)	1008.0 (77.4)	0.110

**Fig. 1:**
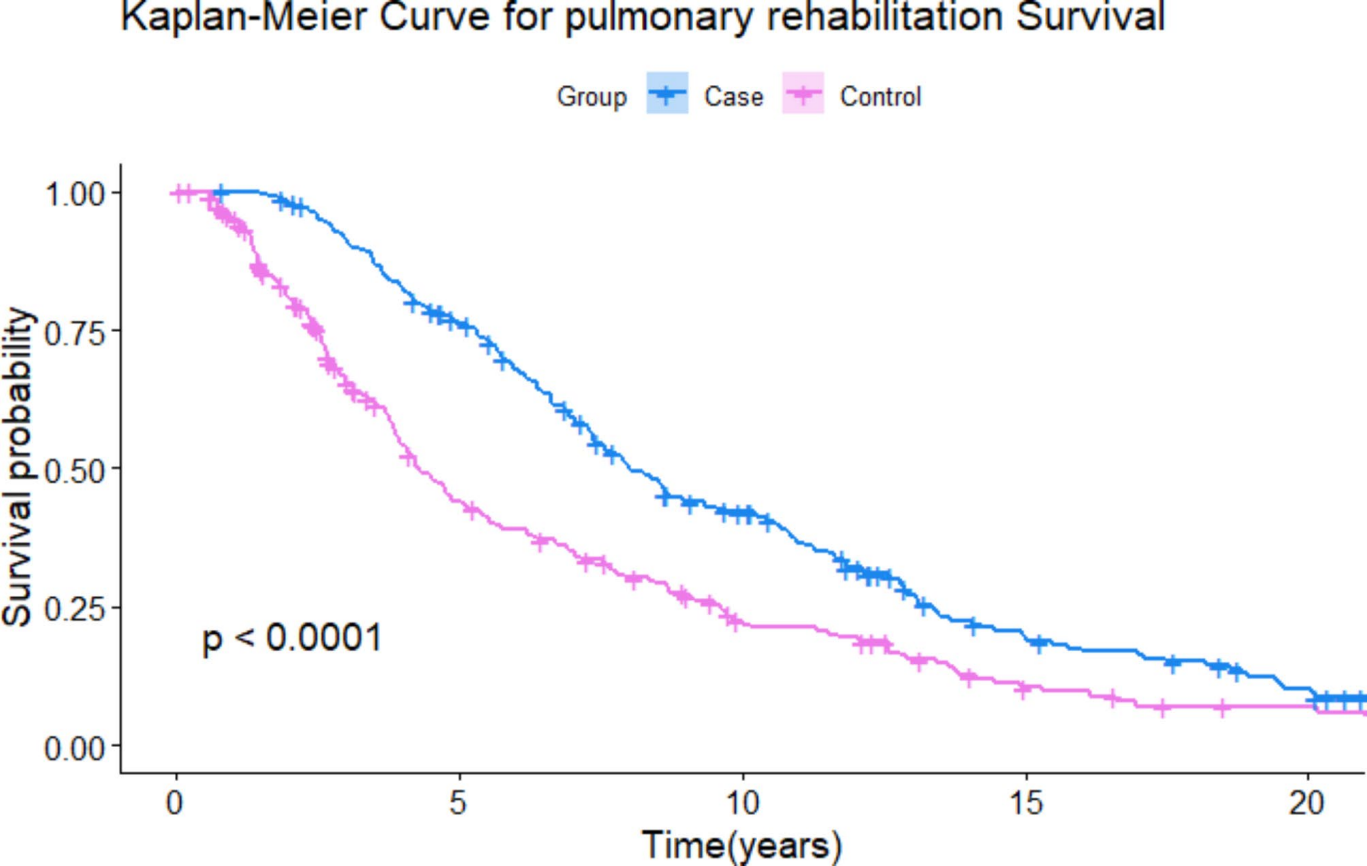
Survival probability of case subjects versus control subjects as a function of time in years

Figure 1 displays the Kaplan-Meier survival analysis depicting the outcomes for both cases and controls. A statistically significant distinction emerged between the two groups according to the results of the log-rank test (*p* < 0.001).

The univariable Cox regression model showed that the control group had a significantly higher risk of death compared to the PR group (Hazard Ratio [HR] = 1.71, 95% CI = 1.39–2.10, *p* < 0.001). After adjusting for age, sex, severity of lung disease, BMI, pack-year smoking, current smoking status and IRSAD, risk of death remained highly significant (HR = 1.64, 95% CI = 1.33–2.35, *p* < 0.001). Males also had an increased risk of death compared to females (HR = 1.85, 95% CI = 1.49–2.30, *p* < 0.001). However, after adjusting for other variables, HR declined to 1.49 suggesting that sex is partly influenced with other variables in the study. The same adjusted model showed that risk of death decreased for each percent increase in FEV_1_ percent predicted (HR = 0.982, 95% CI = 0.976–0.988, *p* < 0.001) and increased for each year increase in age (HR = 1.059, 95% CI = 1.042–1.076, *p* < 0.001). Risk of death decreased for one unit increase in BMI (HR = 0.981, 95% CI = 0.963-1.000, *p* = 0.044) and also decreased for one unit increase in IRSAD (HR = 0.998, 95% CI = 0.997-1.000, *p* = 0.019) after adjusting for other variables. We initially observed a correlation between an increase in pack-year smoking and mortality through univariable analysis. However, this association did not maintain its significance as an independent predictor of mortality when we considered other factors. Notably, the relationship was influenced by sex, with males in the cohort having higher smoking rates (*p* < 0.001). Furthermore, we found that a 1% increase in FEV_1_ percent predicted was associated with a significant reduction of 0.3 units in pack-year smoking (*p* < 0.001). This suggests that the relationship between pack-year smoking and mortality is mediated by both sex and FEV_1_ percent predicted. Current smoking status did not independently predict mortality in either univariable or multivariable models. Table 2 outlines the association between mortality and various variables in both the univariable and multivariable Cox models.

**Table 2:** Hazard ratios and confidence intervals for the Cox regression models

Variables	Univariable			Multivariable		
	**HR**	**CI**	***P*** **value**	**HR**	**CI**	***p*** **value**
Group: control	1.712	1.394–2.101	< 0.001	1.644	1.331–2.030	< 0.001
Age (years)	1.046	1.032–1.061	< 0.001	1.059	1.042–1.076	< 0.001
FEV_1_ (% predicted)	0.983	0.977–0.989	< 0.001	0.982	0.976–0.988	< 0.001
Sex: male	1.847	1.485–2.297	< 0.001	1.487	1.180–1.873	< 0.001
Smoking (Pack-Year)	1.006	1.003–1.008	< 0.001	1.002	0.999–1.005	0.147
Current smoker: yes	1.164	0.825–1.643	0.386	1.138	0.790–1.640	0.486
BMI (Kg/m^2^)	0.963	0.947–0.980	< 0.001	0.981	0.963-1.000	0.044
IRSAD	0.999	0.998-1.000	0.167	0.998	0.997-1.000	0.019

The concordance index of the multivariable Cox model was 0.72 indicating that the model had a moderate level of discriminatory power in predicting mortality. The univariable model assessing the effect of PR on mortality demonstrated a concordance index of 0.60, indicating a comparatively lower level of predictive accuracy. This improvement in the concordance index in the multivariable model indicates that its predictive accuracy was enhanced by considering additional factors.

We conducted a similar multivariable Cox regression analysis on a subgroup of patients with COPD, consisting of 210 cases and 210 controls. Within this model, the factors predicting mortality independently were PR enrollment, age, and FEV_1_ percent predicted. Notably, the adjusted HR for mortality in the control group closely mirrored the HR calculated in the primary cohort analysis (HR = 1.70, CI = 1.37–2.07, *p* < 0.001).

14% of the initial cases enrolled in PR twice, while 0.7% enrolled three times. However, these additional enrollments did not result in any significant survival benefit compared to the other cases (HR = 0.998, 95% CI = 0.683–1.460, *p* = 0.992).

## Discussion

Our findings show that patients with clinically stable chronic respiratory disease who participated in a PR program had a significantly lower mortality rate compared to disease matched control patients who did not undertake PR, after adjusting for age, sex, severity of lung disease, BMI, pack-year smoking, current smoking status and IRSAD.

These findings support the limited evidence from previous PR trials in stable patients. In a controlled trial of 119 patients with COPD, Ries et al. (1995) [17] reported 85% survival at three years and 67% survival at six years in the PR group compared with 74% survival at three years and 56% survival at six years in the education only control group. Griffiths et al. (2000) [18] randomized 200 patients with stable chronic respiratory disease to a six-week multidisciplinary PR program or usual medical care and compared survival at 12 months, reporting 95% in the PR group and 90% in the usual care group. Differences between groups were not statistically significant in either trial. Reported survival rates in these PR groups are similar to several cohort studies reporting one-year survival 91–95% [19, 20] and three-year survival 80–85% [20, 21] following completion of PR.

In a large retrospective analysis of over 1500 patients with stable COPD, completion of PR (54%) was associated with significantly increased survival time up to 10 years compared with failure to complete PR (46%) [22]. Similarly, in a multicentre retrospective study of patients with ILD [23], participation in more than 80% of a PR program was associated with a lower risk of death (HR = 0.67) within four years compared with patients who participated in less than 80%. Based on these findings, it is likely that our analyses have underestimated the survival benefit of PR as we included all patients from the PR database who met the inclusion criteria irrespective of participation or completion rates.

There are methodological limitations with this retrospective cohort study. The PR and control groups were selected from two separate databases rather than being randomly allocated, introducing a selection bias. Patients referred to PR might have a physician who is more proactive in managing their disease, and those who agree to participate in PR may be more receptive to and capable of managing their condition. These factors could influence both PR participation and potentially affect survival rates. However, we do not have information on the specific reasons why some patients were referred to PR while others were not, or why some who were referred chose not to attend. Moreover, some individuals in the control group may have participated in PR after 2007 or at other hospitals, which could also influence the outcomes observed.

Our data on program adherence was limited to whether patients enrolled in the program, meaning they attended at least one session, and whether they participated in a post-program review. Our approach of including all enrolled patients was conservative and aimed to reflect an intention-to-treat methodology in our retrospective study. Although we are not suggesting that attending just one session impacts long-term survival, including all participants who attended at least one session may have led to an underestimation of the benefits.

Additionally, we did not investigate cause of death either in cases or in controls, so the number of deaths that were respiratory related is unknown. There were unreported variables such as baseline six-minute walk distance, utilization of long-term oxygen therapy and co-morbidities that potentially impact survival but could not be included in the analyses and may therefore impact the accuracy of the Cox model.

## Conclusion

Despite these methodological limitations, our findings suggest that PR may have a mortality benefit for stable outpatients with chronic respiratory disease. This benefit also extends to a subgroup of patients with COPD and the observed benefits may be underrepresented because our study followed an intention-to-treat approach. Our results reinforce the importance of PR in comprehensive patient management of chronic respiratory disease.

## Data Availability

The datasets used and analyzed during the current study are available from the corresponding author on reasonable request.

## Abbreviations

BMI: Body Mass Index
CI: Confidence Interval
COPD: Chronic Obstructive Pulmonary Disease
FEV_1_: Forced Expiratory Volume in one second
HR: Hazard Ratio
ILD: Interstitial Lung Disease
IQR: Inter Quartile Range
IRSAD: Index of Relative Socio-economic Advantage and Disadvantage
PR: Pulmonary Rehabilitation
SD: Standard Deviation
UR: Unique Record
